# Survival-Associated Metabolic Genes in Human Papillomavirus-Positive Head and Neck Cancers

**DOI:** 10.3390/cancers12010253

**Published:** 2020-01-20

**Authors:** Martin A. Prusinkiewicz, Steven F. Gameiro, Farhad Ghasemi, Mackenzie J. Dodge, Peter Y. F. Zeng, Hanna Maekebay, John W. Barrett, Anthony C. Nichols, Joe S. Mymryk

**Affiliations:** 1 Department of Microbiology and Immunology, The University of Western Ontario, London, ON N6A 3K7, Canada; 2 Department of Surgery, The University of Western Ontario, London, ON N6A 3K7, Canada; 3 Department of Otolaryngology, Head & Neck Surgery, The University of Western Ontario, London, ON N6A 3K7, Canada; yzeng2023@meds.uwo.ca (P.Y.F.Z.);; 4 Department of Oncology, The University of Western Ontario, London, ON N6A 3K7, Canada; 5 London Regional Cancer Program, Lawson Health Research Institute, London, ON N6C 2R5, Canada

**Keywords:** human papillomavirus, head and neck cancer, cancer metabolism, glycolysis, cellular respiration, TCGA

## Abstract

Human papillomavirus (HPV) causes an increasing number of head and neck squamous cell carcinomas (HNSCCs). Altered metabolism contributes to patient prognosis, but the impact of HPV status on HNSCC metabolism remains relatively uncharacterized. We hypothesize that metabolism-related gene expression differences unique to HPV-positive HNSCC influences patient survival. The Cancer Genome Atlas RNA-seq data from primary HNSCC patient samples were categorized as 73 HPV-positive, 442 HPV-negative, and 43 normal-adjacent control tissues. We analyzed 229 metabolic genes and identified numerous differentially expressed genes between HPV-positive and negative HNSCC patients. HPV-positive carcinomas exhibited lower expression levels of genes involved in glycolysis and higher levels of genes involved in the tricarboxylic acid cycle, oxidative phosphorylation, and β-oxidation than the HPV-negative carcinomas. Importantly, reduced expression of the metabolism-related genes *SDHC*, *COX7A1*, *COX16*, *COX17*, *ELOVL6*, *GOT2*, and *SLC16A2* were correlated with improved patient survival only in the HPV-positive group. This work suggests that specific transcriptional alterations in metabolic genes may serve as predictive biomarkers of patient outcome and identifies potential targets for novel therapeutic intervention in HPV-positive head and neck cancers.

## 1. Introduction

As of 2018, head and neck squamous cell carcinomas (HNSCC), namely cancers of the oral cavity, oropharynx, nasopharynx, larynx, and hypopharynx, had the 8th highest combined incidence rate and the 5th highest 5-year prevalence as interpreted from GLOBOCAN data [1,2]. This translates to 834,860 new head and neck cancers per year and 2,164,271 active head and neck cancers within the past five years worldwide [1,2]. Recent incidence rates of some oropharyngeal cancers, such as those of the tonsils and base of the tongue, have been rapidly increasing due to high-risk human papillomavirus (HPV) infection [3]. Infection by specific high-risk HPVs, such as HPV16, was only recognized as a contributing factor for oropharyngeal cancer by the International Agency for Research on Cancer in 2003 [4]. However, the number of oropharyngeal cancers caused by HPV has risen at epidemic rates over the last decades in many parts of the world [5], while the number of HNSCCs caused by exposure to mutagens from excessive smoking and drinking has been decreasing [6].

HPV-positive (HPV+) HNSCCs are distinct from their HPV-negative (HPV-) counterparts from a molecular perspective, with characteristic genetic, epigenetic, and protein expression profiles [7,8,9]. In addition, patient outcomes are generally far more favorable for HPV+ than HPV- HNSCC [10]. The underlying molecular reasons for this difference are not entirely clear. However, approximately 10% of all HPV+ HNSCC patients still succumb to their disease [11]. Identification of prognostic markers predicting favorable survival outcomes in patients could allow for treatment deintensification, thereby avoiding potential lifelong complications from unnecessarily aggressive treatments. Alternatively, identification of cellular pathways contributing to poor prognosis could lead to the development of new effective therapies for those not responding to the current standard of care. 

Altered metabolism is a cancer hallmark that was recognized decades ago with the discovery of the Warburg effect, also known as aerobic glycolysis [12]. Aerobic glycolysis involves an upregulation of glycolysis despite the presence of ample oxygen for efficient cellular respiration. Many tumours exhibit this metabolic phenotype, as it provides rapid energy and an ample supply of precursors for macromolecule biosynthesis. Tumours can also rely on cellular respiration, often via glutaminolysis, which is the breakdown of glutamine into intermediates of the tricarboxylic acid (TCA) cycle. [13]. TCA intermediates can be funneled off for macromolecule biosynthesis. Many viruses are known to extensively modulate cellular metabolic processes to facilitate infection [14]. These changes can include similar tumour-associated metabolic changes as described above. Infection with HPV has been shown to phenocopy cancer-like metabolic changes that are maintained in HPV+ HNSCC [15]. Examination of how the metabolic phenotype differs between HPV+ and HPV- HNSCC could lead to the identification of targetable metabolic changes and potential new treatment options that are specific for either HPV+ HNSCCs or HPV- HNSCCs. Admittedly, cancer metabolism is complex as it impinges on a variety of other cellular processes and can vary across an individual tumour [16]. In addition, tumours can be highly adaptive to metabolic perturbations [16]. Identifying multiple metabolic targets that are specific to a cancer type from a large dataset that contains information from an extensive number of tumours, such as The Cancer Genome Atlas (TCGA), is an ideal step towards selecting or generating useful anti-metabolic cancer therapeutics. 

In this study, we used RNA-seq data from over 500 HNSCC primary tumour samples from the TCGA to comprehensively compare the expression of genes across key cellular metabolic pathways between HPV+, HPV-, and normal-adjacent control tissues. Expression of a number of metabolic genes were significantly altered in HPV+ versus HPV- HNSCC. Specifically, genes involved in glycolysis were expressed at lower levels in HPV+ compared to HPV- HNSCC. In contrast, genes involved in the TCA cycle, oxidative phosphorylation, and β-oxidation exhibited higher expression in HPV+ samples compared to their HPV- counterparts. Importantly, we identified that low expression of multiple metabolic genes—*SDHC*, *COX7A1*, *COX16*, *COX17*, *ELOVL6*, *GOT2*, and *SLC16A2*—correlated with markedly improved patient survival in HPV+, but not HPV- HNSCC. The products of these genes could potentially be exploited as targets for therapeutic intervention. Furthermore, the low expression of these seven genes appears useful in predicting improved overall survival in HPV+ HNSCC and could serve as biomarkers of patient outcome.

## 2. Results

### 2.1. Expression of Pathway-specific Metabolic Genes Were Altered Between HPV+, HPV-, and Normal Control Samples from The TCGA HNSC Cohort

**Figure 1:**
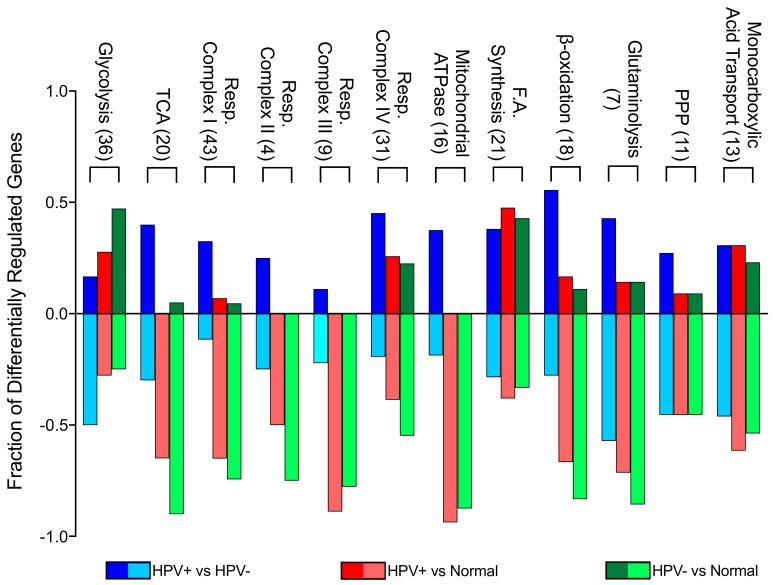
Genes differentially expressed between HPV-positive (HPV+) head and neck squamous cell carcinomas (HNSCC), HPV-negative (HPV-) HNSCC, and normal control tissues by metabolic pathway. The y-axis reflects the proportion of genes that are up or downregulated in a given pathway comparison. For example, the positive fraction of genes reflects the proportion of genes upregulated in the first group (e.g., HPV+) when compared to the second group (e.g., HPV-). The negative fraction of genes reflects the proportion of genes downregulated in the same comparison. Blue = HPV+ tissue vs HPV- tissue comparison; Red = HPV+ tissue vs normal control tissue comparison; Green = HPV- tissue vs normal control tissue comparison. Numbers in brackets denote total number of genes analyzed from each pathway. Abbreviations: TCA, tricarboxylic acid cycle; Resp., respiratory; F.A., fatty acid; PPP, pentose phosphate pathway.

In order to identify differences in metabolic gene expression between HPV+ and HPV- HNSCC, we analyzed the TCGA Illumina HiSeq RNA expression dataset from the HNSC cohort for expression of 229 metabolic genes in central metabolic pathways (Supplementary Table S1). This clinical cohort is comprised of 73 HPV+, 442 HPV-, and 43 normal control samples with available RNA-seq data. Significant differences were seen in a subset of genes in each pathway between HPV+ and HPV- HNSCC. In addition, significant changes were observed between either HPV+ HNSCC or HPV- HNSCC and normal control tissue. To simplify interpretation of these differences, we plotted the fraction of genes in each pathway that were significantly different for each pairwise comparison (Figure 1). 

This analysis illustrates that glycolytic genes in HPV- HNSCC tissue are more upregulated in comparison to normal tissue than in HPV+ HNSCC when compared to normal tissue. This is particularly evident between HPV+ HNSCC and HPV- HNSCC tissues, since most glycolytic genes are downregulated in HPV+ HNSCC when compared to HPV- HNSCC. However, genes involved in the TCA cycle, oxidative phosphorylation, and β-oxidation were downregulated in both HPV+ and HPV- HNSCC in comparison to normal control tissue. This means that, despite differences in metabolic gene expression between HPV+ and HPV- HNSCC, the metabolism of both types of tumours could resemble the Warburg effect, with lower cellular respiration rates compared to normal control tissue. When compared to one another, HPV+ HNSCC had generally higher expression of these genes than HPV- HNSCC. Other metabolic pathways appeared to have a more similar split between upregulated and downregulated genes in all comparisons, suggesting that the presence of HPV may have minimal impact on transcriptionally mediated changes in these pathways. Overall, it appears that HPV+ HNSCC may be more reliant on cellular respiration than HPV- HNSCC.

### 2.2. Low Expression of Genes Encoding Multiple Components of the Mitochondrial Electron Transport Chain Are Associated with Improved Patient Survival in HPV+ HNSCC

Tumour-associated metabolic alterations have functional consequences that impact disease progression, response to therapy, and patient survival [13]. We dichotomized the expression data for each of the 229 metabolic genes by median expression and calculated the impact of high versus low expression on overall patient survival for HPV+ HNSCC patients, as well as HPV- HNSCC patients (Supplementary Table S2). We identified seven genes that were significantly associated with patient survival in HPV+, but not HPV- HNSCC patients. These genes were *SDHC*, part of the mitochondrial respiratory complex II; *COX7A1*, *COX16*, and *COX17*, all part of the mitochondrial respiratory complex IV; *ELOVL6*, involved in fatty acid elongation; *GOT2*, involved in amino acid metabolism; and *SLC16A2* (also known as *MCT8*), which encodes a thyroid hormone transporter. We performed a pathway enrichment analysis of our seven significant genes utilizing a PANTHER overrepresentation test which indicated that 5 of these 7 genes were significantly associated with the mitochondria (*p* = 5.10 x 10^-5^; FDR = 1.46 x 10^−2^). These genes were *SDHC*, *COX7A1*, *COX16*, *COX17*, and *GOT2*. Detailed studies of the relative expression of each of these genes in HPV+, HPV- and normal control tissue, as well as their association with overall patient survival are presented below.

**Figure 2:**
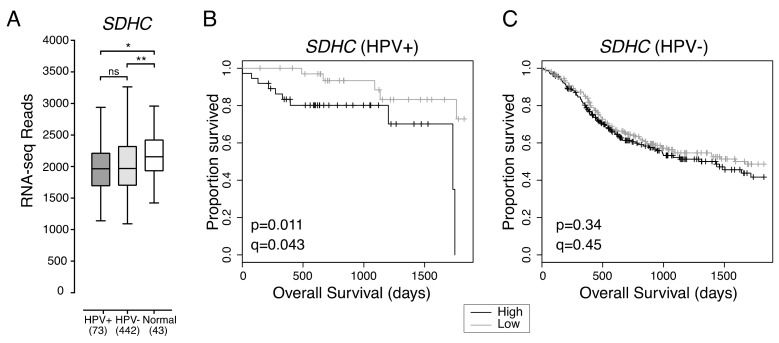
Low expression of *SDHC* is associated with favorable survival outcomes in HPV+ HNSCC. (**A**) Transcript levels of *SDHC* across all HNSCC tissues samples and normal control tissues. Bracketed numbers refer to the sample size of each group. Overall five-year survival outcomes in (**B**) HPV+ HNSCC and (**C**) HPV- HNSCC patients dichotomized by *SDHC* expression. *p* = Two-sided log-rank test, *q* = Benjamini-Hochberg FDR method. Gray = low transcript expression, Black = high transcript expression. * *p* ≤ 0.05, ** *p* ≤ 0.01, ns (not significant).

Previous studies indicate that HPV+ HNSCC is more reliant on oxidative phosphorylation as an energy source than HPV- HNSCC [15]. Oxidative phosphorylation requires electron transport via mitochondrial cellular respiratory complexes I-IV [17]. Reduced expression of *SDHC*, which encodes a component of the mitochondrial cellular respiration complex II, correlated with increased HPV+ HNSCC patient survival (Figure 2). 

Overall expression of *SDHC* was not significantly different between HPV+ and HPV- HNSCC, and both types of HNSCC expressed lower levels of *SDHC* than normal control tissue (Figure 2A). HPV+ HNSCC patients with tumours exhibiting low *SDHC* expression had better overall five-year survival outcomes than HPV+ HNSCC patients with tumours exhibiting high *SDHC* expression (*p* = 0.011, FDR = 0.043) (Figure 2B). However, *SDHC* expression was not correlated with improved patient survival in HPV- HNSCC (*p* = 0.34, FDR = 0.45) (Figure 2C).

**Figure 3:**
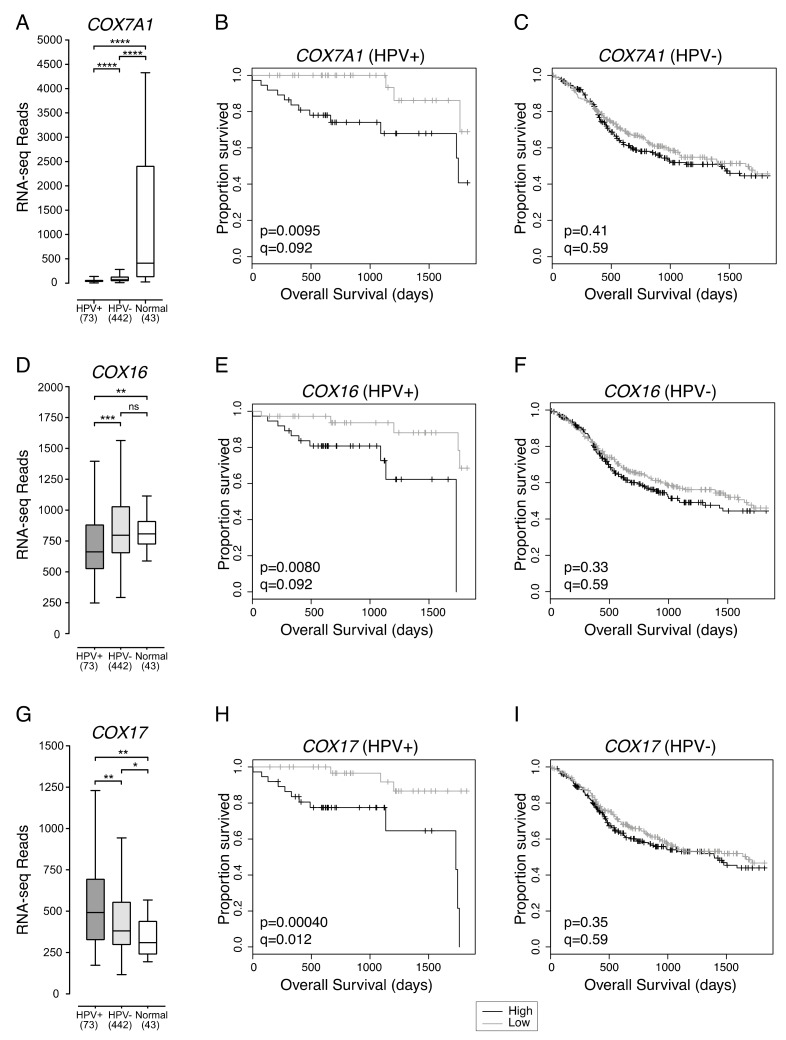
Low expression of three mitochondrial respiration complex IV genes in HPV+ HNSCC is associated with improved survival. Expression of (**A**) *COX7A1*, (**D**) *COX16*, and (**G**) *COX17* in HNSCC tissues samples and normal control tissues. Overall 5-year survival outcomes in HPV+ HNSCC patients and HPV- HNSCC dichotomized by median (**B**,**C**) *COX7A1* expression, (**E**,**F**) *COX16* expression, and (**H**,**I**) *COX17* expression. *p* = Two-sided log-rank test, *q* = Benjamini-Hochberg FDR method. Gray = low transcript expression, Black = high transcript expression. * *p* ≤ 0.05, ** *p* ≤ 0.01, *** *p* ≤ 0.001, **** *p* ≤ 0.0001, ns (not significant).

Consistent with the possibility that HPV+ HNSCCs are more reliant on cellular respiration than HPV- HNSCCs, three genes encoding components of the mitochondrial cellular respiration complex IV were also correlated with HPV+ HNSCC patient survival (Figure 3). These genes were *COX7A1* and *COX17*, which encode structural components of complex IV, and *COX16*, whose product is involved in complex IV assembly. 

*COX7A1* expression was significantly lower in both HPV+ and HPV- HNSCC samples compared to normal control tissues (Figure 3A). In addition, HPV+ HNSCC had significantly lower expression of *COX7A1* than HPV- HNSCC (Figure 3A). Overall survival of patients with HPV+ (Figure 3B) or HPV- HNSCC (Figure 3C) were dichotomized based on median *COX7A1* expression. We found that low expression of *COX7A1* was correlated with favourable survival outcomes in patients with HPV+ (*p* = 0.0095, FDR = 0.092) (Figure 3B), but not HPV- HNSCC (*p* = 0.41, FDR = 0.59) (Figure 3C). 

Compared to normal control tissue, *COX16* expression was lower in HPV+, but not in HPV- HNSCC (Figure 3D). *COX16* expression was also significantly lower in HPV+ compared to HPV- HNSCC (Figure 3D). HPV+ and HPV- HNSCC samples were dichotomized based on median *COX16* expression. Low levels of *COX16* expression were correlated with improved survival in patients with HPV+ (*p* = 0.0080, FDR = 0.092) (Figure 3E), but not in patients with HPV- HNSCC (*p* = 0.33, FDR = 0.59) (Figure 3F). 

In contrast to *COX7A1* and *COX16*, *COX17* expression was higher in HPV+ than HPV- HNSCC (Figure 3G). Expression of *COX17* across HPV+ and HPV- HNSCC samples was significantly higher than in normal control tissues (Figure 3G). However, the same association between low expression of *COX17* and better overall 5-year patient survival was observed for patients with HPV+ HNSCC (*p* = 0.00040, FDR = 0.012) (Figure 3H), but not patients with HPV- HNSCC (*p* = 0.35, FDR = 0.59) (Figure 3I).

### 2.3. Low Expression of ELOVL6, Involved in Fatty Acid Synthesis, Is Associated with Better Overall Survival in Patients with HPV+ HNSCC

**Figure 4:**
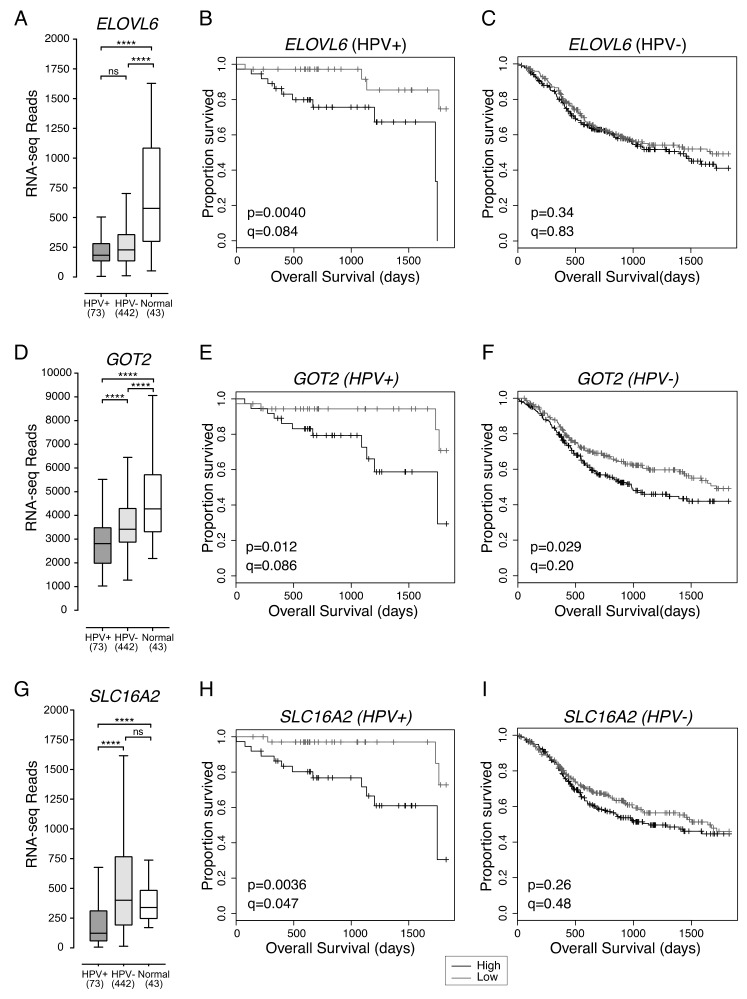
Low expression of *ELOVL6*, *GOT2* and *SLC16A2* is associated with improved patient survival in HPV+ HNSCC. Expression of (**A**) *ELOVL6*, (**D**) *GOT2*, and (**G**) *SLC16A2* in HPV+, HPV- HNSCC tissue samples and normal control tissues. Overall five-year survival outcomes in HPV+ HNSCC and HPV- HNSCC patients dichotomized by median (**B**,**C**) *ELOVL6* expression, (**E**,**F**) *GOT2* expression, and (**H**,**I**) *SLC16A2* expression. *p* = Two-sided log-rank test and *q* = Benjamini-Hochberg FDR method. Gray = low transcript expression, Black = high transcript expression. **** *p* ≤ 0.0001, ns (not significant).

*ELOVL6* expression in HPV+ HNSCC was not significantly different from HPV- HNSCC. However, both HPV+ and HPV- HNSCC had significantly lower overall levels of *ELOVL6* expression than normal control tissues (Figure 4A). HPV+ HNSCC samples were dichotomized based on median *ELOVL6* expression. HPV+ patients with tumours expressing low levels of *ELOVL6* had significantly better five-year overall survival than patients with tumours expressing high levels of *ELOVL6* (*p* = 0.0040, *q* = 0.084) (Figure 4B). Reduced *ELOVL6* expression was not correlated with altered survival in HPV- HNSCC patients (*p* = 0.34, *q* = 0.83) (Figure 4C).

### 2.4. Low Expression of GOT2, Involved in Amino Acid Metabolism, Is Associated with Better HPV+ HNSCC Patient Survival

*GOT2* expression was significantly lower in HPV+ than HPV- HNSCC or normal control tissues and significantly lower in HPV- HNSCC than normal control tissues (Figure 4D). The high normalized RNA-seq read levels for *GOT2* suggest that it is abundantly expressed in normal head and neck tissues. HPV+ HNSCC samples were dichotomized based on median *GOT2* expression. Low expression of *GOT2* was associated with better five-year overall survival outcomes in patients with HPV+ HNSCC (*p* = 0.012, *q* = 0.086; Figure 4E). Although low *GOT2* expression appeared to be significantly correlated with favourable patient survival in HPV- HNSCC (*p* = 0.029; Figure 4F), it lost its significance after correcting for FDR (*q* = 0.20). 

### 2.5. Low Expression of SLC16A2, a Thyroid Hormone Transporter, in HPV+ HNSCC Is Associated with Better Overall Survival

Increased expression of the monocarboxylic acid transporter family member, *SLC16A1* (*MCT1*) was recently reported to be associated with poor survival outcomes in HNSCC [15]. Although, expression of *SLC16A1* was significantly higher than normal head and neck tissues for both HPV+ and HPV- HNSCC, and expression of *SLC16A1* was higher in HPV- HNSCC than HPV+ HNSCC, the impact of differential expression of *SLC16A1* on overall survival in either HPV+ or HPV- HNSCC was not significant (*p* > 0.05). Of the various family members, only expression of *SLC16A2* (*MCT8*), whose main function is thyroid hormone transport, appeared to be associated with altered overall survival (Figure 4G–I). 

Expression of *SLC16A2* was significantly lower in HPV+ HNSCC when compared to either HPV- HNSCC or normal control tissues (Figure 4G). HPV+ HNSCC samples were dichotomized based on median *SLC16A2* expression. Again, low expression of *SLC16A2* was associated with improved patient survival in HPV+ HNSCC patients (*p* = 0.0036, FDR = 0.047) (Figure 4H), but not in patients with HPV- HNSCC (*p* = 0.26, FDR = 0.48) (Figure 4I). 

### 2.6. COX16, COX17, and SLC16A2 Are Independently Correlated with Favourable Survival Outcomes in HPV+ HNSCC

To determine the extent that each of the HPV+ HNSCC survival-associated genes could influence patient outcomes, we generated a hazard ratio (HR) for each gene and a variety of clinical variables by univariate analysis (Table 1). Each HR describes the relative increase in risk of death for the first variable x vs y [18]. As expected, the HR for each metabolic gene was significantly below 1, indicating a greatly reduced risk of death. In contrast, a comparison of the oral cavity vs the oropharynx subsites for HPV+ HNSCC generated a hazard ratio of 2.82, indicating that HPV+ HNSCC in the oral cavity is associated with a 2.82x increased risk of mortality compared to oropharynx.

As the contribution of these genes to overall survival might not be independent of one another, we also analyzed the relationship between survival and gene expression for all survival-associated metabolic genes and clinical variables concurrently by multivariate analysis (Table 1). The hazard ratios for COX16, COX17, and SLC16A2 remained significant, indicating that low expression of each of these genes is a significant, and potentially independent, contributor to overall survival. COX7A1 had a minor contribution to survival in this model. The multivariate model also included subsite (oral cavity vs. oropharynx) and HPV type as significant contributing factors to survival as previously reported in the literature [19,20,21,22].

**Figure 5:**
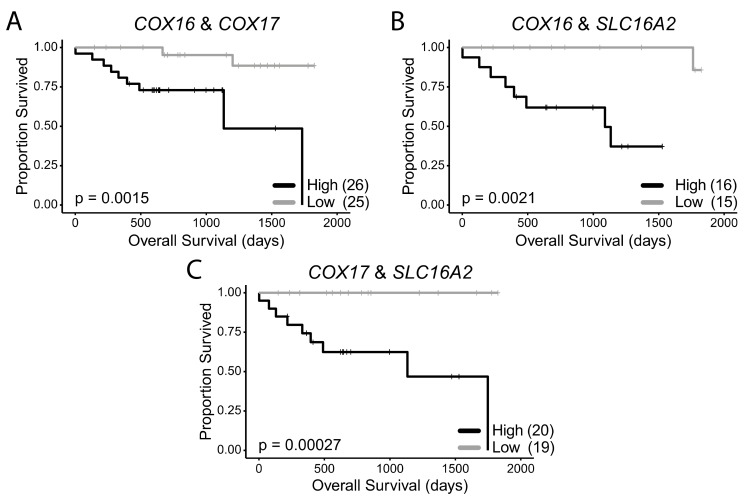
HPV+ HNSCC patient survival stratified by double low or double high expression of survival-associated metabolic genes. (**A**) *COX16* and *COX17*, (**B**) *COX16* and *SLC16A2,* (**C**) *COX17* and *SLC16A2*. Comparisons made with a two-sided log-rank test. Gray = low transcript expression, Black = high transcript expression. Bracketed value indicates number of HPV+ HNSCCs with double high or double low expression for genes of interest.

To test whether concurrent low expression of these genes had an additive effect on survival, we stratified the HPV+ HNSCCs into groups, based on high expression or low expression for a combination of any two of these seven survival-associated genes. When survival of HPV+ HNSCC patients with low expression of both *COX16* and *COX17* in their tumours was compared to survival of patients with high expression of both genes, survival was significantly greater in the *COX16* and *COX17* double low expression group (*p* = 0.0015) (Figure 5A). In patients with low expression of both *COX16* and *SLC16A2*, survival was virtually 100% until approximately 4.75 years and significantly better (*p* = 0.0021) than samples expressing high levels of both genes (Figure 5B). In patients with low expression of both *COX17* and *SLC16A2* (Figure 5C), survival was 100%, which was significantly higher than patients expressing high levels of both *COX17* and *SLC16A2* (*p* = 0.00027; Figure 5C). 

Supplementary Figure S1 shows that there was no significant correlation between the expression of *SLC16A2* and *COX16* or *SLC16A2* and *COX17*. This provides further evidence that the improved survival associated with low expression for each of these genes may occur independently of one another. Additionally, the multivariate analysis indicates that *COX16* and *COX17* independently contribute to survival despite the correlation between these two genes (Supplementary Figure S1).

## 3. Discussion

Our analysis identified many changes in expression of metabolism-associated genes between HPV+ and HPV- HNSCC when compared to normal control tissues. The expression of seven genes was predictive of survival for HPV+ HNSCC patients. In each case, reduced expression correlated with improved survival, suggesting that reduced tumour cell metabolism is prognostically favorable. None of these genes were associated with altered survival in HPV- HNSCC, reinforcing the concept that HPV+ and HPV- HNSCC are distinct tumour entities [7,8,9,23]. This is not unexpected, as E6 from HPV16 and HPV18 can increase the expression of mitochondrial cellular respiration genes in a head and neck cancer cell line [24], which matches our observation of increased expression of these genes in HPV+ HNSCCs when compared to HPV- HNSCCs. In addition, E6 and E7 may also be responsible for perturbing glycolysis in HPV+ cervical cancer cells [25,26], which could explain our observation of increased expression of glycolytic genes in HPV+ HNSCC as compared to normal control tissues. Interestingly, most of the survival-associated genes we identified in our study can be inhibited by small molecule inhibitors as outlined below. 

As shown in our results, limiting *SDHC* may serve as a unique target in virally transformed HPV+ HNSCCs. *SDHC* encodes part of mitochondrial respiratory complex II, for which a few selective inhibitors exist. α-tocopheryl succinate (α-TOS) is a vitamin E analogue, which has selective growth inhibitory properties for some human cancer cells [27]. α-TOS can induce apoptosis by increasing the levels of reactive oxidative species (ROS), triggering stress response pathways [27]. α-TOS is also effective at inhibiting tumour growth [28] in in vivo xenograft mouse models. Interestingly, α-TOS inhibited growth of several HNSCC cell lines in vitro and in vivo [29]. Given that all of these experiments were done using HPV- HNSCC, α-TOS may be even more toxic to HPV+ HNSCCs based on the correlation between *SDHC* expression and HPV+ HNSCC survival outcomes we observed.

Specific small molecule inhibitors for both the *COX7A1* and *COX16* gene products have not yet been identified. However, as both are part of mitochondrial respiratory complex IV, it is possible that the complex IV inhibitors ADDA 5 [30] and tetrathiomolybdate [31] could prove useful to phenocopy any metabolic effects associated with low gene expression, promoting enhanced survival in HPV+ HNSCC. It is also important to note that COX16 is an inhibitor of p53 activity, which means that non-mitochondrial functions of COX16 should not be discounted [32]. E6 has been shown to inhibit expression of COX16 [33], which may contribute to the lower levels observed in HPV+ versus HPV- HNSCC (Figure 3D). MitoBloCK-6 is an inhibitor of mitochondrial respiratory complex IV that specifically targets the COX17 protein [34]. Whether inhibition of cellular respiration is less effective in HPV- HNSCCs because they are already more oxidatively stressed than HPV+ HNSCCs [35], perhaps as a result of being less adapted to utilize cellular respiration, is an open question. 

Two inhibitors of ELOVL6 were able to reduce the fatty acid composition of hepatocytes and the liver in a murine model of obesity [36,37], but the effects of these compounds on cancer cells have not been explored. Another potential druggable target to influence ELOVL6 expression is ATP citrate lyase (ACLY), which has a wide variety of inhibitors [38]. Expression of ELOVL6 has been shown to decrease concurrently with ACLY inhibition [39]. Whether ELOVL6 inhibition would reduce the growth of HPV+ HNSCC cell lines remains to be examined.

In our study, we found that low expression of *GOT2* was associated with statistically significant survival in both groups (p < 0.05). However, only expression of *GOT2* in our HPV+ HNSCC group met the FDR cut-off of *q* = 0.1. This means that while *GOT2* may be important for survival outcomes in both HPV+ and HPV- HNSCC, it is likely that it has a more substantial contribution to patient survival in HPV+ HNSCC. In breast cancer, sensitivity to a nucleotide synthesis inhibitor, methotrexate, has been linked to high *GOT2* expression [40]. This is likely due to the function of GOT2 in providing aspartate for nucleotide biosynthesis [40]. It is possible that HPV+ HNSCCs, or potentially any HNSCCs expressing high levels of *GOT2*, may be sensitive to methotrexate, but this remains to be explored.

*SLC16A2* encodes a plasma membrane T3/T4 transporter. Once inside the cell, T3/T4 can bind nuclear and mitochondrial-localized thyroid hormone receptors, which are key regulators of mitochondrial biogenesis [41]. As HPV+ HNSCC may be more reliant on cellular respiration than HPV- HNSCC, it is possible that inhibiting SLC16A2-mediated thyroid hormone transport across the plasma membrane could preferentially inhibit ATP generation in HPV+ HNSCCs. Some tyrosine kinase inhibitors (TKIs), such as sunitinib, imatinib, dasatinib, and bosutinib, may inhibit SLC16A2 [42]. These TKIs are already employed to treat a wide variety of cancers and are being evaluated for the treatment of HNSCC [43]. As such, they may be especially suitable for the treatment of HPV+ HNSCCs expressing *SLC16A2* at high levels. We extracted data from our previous study of 27 HNSCC cell lines (6 HPV+ and 21 HPV- HNSCC cell lines) examining the effects of a variety of agents on cell growth and proliferation, including the TKI inhibitors mentioned above [44]. Of the TKIs tested, dasatinib was selectively cytotoxic to HPV+, but not HPV- HNSCC cell lines (Supplementary Figure S2), suggesting that it could be used as a treatment for HPV+ HNSCC that expresses high levels of *SLC16A2*. A flavonoid, silychristin, also inhibits SLC16A2 [45], but has not been studied in cancer models. Other antithyroid hormones used to treat hyperthyroidism, such as carbimazole, methimazole, and potassium perchlorate, could preferentially inhibit HPV+ HNSCC by mimicking inhibition of the SLC16A2 transporter. In one study, methimazole was used to experimentally treat patients with end-stage solid tumours and resulted in improved survival [46]. High levels of thyroid hormone can promote proliferation of some cancers [47], and thyroid hormone mimetics which function as antagonists, such as tetraiodothyroacetic acid (tetrac) and triiodothyroacetic acid (triac) appear to exhibit an antiproliferative effect on breast cancer [47] and T cell lymphomas [48].

While our univariate analysis of all seven genes confirmed that their expression was significantly correlated with survival, our multivariate analysis indicated that *COX16*, *COX17*, and *SLC16A2* were independently associated with overall survival. This suggested that the collective changes in the expression of these three genes could be a powerful predictor of clinical outcome. This was supported by our observation that the overall survival for the group of patients exhibiting simultaneously low expression of any two of these genes was far better than those simultaneously expressing high levels. Thus, expression of these genes may have prognostic utility. Furthermore, *COX16*, *COX17*, and *SLC16A2* may represent attractive therapeutic targets for HPV+ HNSCC that warrant further exploration, especially considering that treatment with specific inhibitors may phenocopy the effects of low expression of these metabolic genes. 

It is important to be cognizant of the limitations to this kind of study. One limitation to this study was the lack of protein data in the TCGA to corroborate our HPV+ HNSCC mRNA expression data. This is an important consideration, as levels of protein expression do not necessarily mirror mRNA expression [49]. As with any high throughput dataset, batch effects that result from processing could be reflected in the data [50]. In addition, the RNA-seq data contained within the TCGA reflects average mRNA expression within the whole tumour and does not identify expression differences between the various tumour cells as could be obtained from single-cell RNA sequencing platforms [51,52]. However, the bulk of the tissue that was sequenced is of tumour origin [52]. Also, it is important to be aware that the TCGA contains HNSCC data from a single, albeit high quality, cohort and it would be useful to validate these genes of interest in other cohorts in the future. Finally, the concept of biomarker identification itself has its own caveats. Specifically, all of the survival-associated genes we identified in this study are only correlated with survival, which does not equal causation [53]. In addition, as with all studies of this type, there exists the possibility that these correlations are due to chance or occur as the result of another confounder [53]. However, despite these limitations, the seven metabolic genes identified in this study provide an interesting starting point for considering the metabolic differences between HPV+ and HPV- HNSCC as new prognostic markers or potential targets for therapy.

## 4. Materials and Methods 

### 4.1. Data Collection

Level 3 RNA-seq by Expectation Maximization normalized Illumina HiSeq RNA expression data (build 2016012800) for the TCGA HNSC cohort, was downloaded from the Broad Genome Data Analysis Centers Firehose server (https://gdac.broadinstitute.org/). Patient survival data for the TCGA HNSC cohort, as reported by the Pan-Cancer Atlas [54], was downloaded from: https://www.cell.com/cms/10.1016/j.cell.2018.02.052/attachment/f4eb6b31-8957-4817-a41f-e46fd2a1d9c3/mmc1.xlsx.

### 4.2. RNA Expression Comparisons

RNA-seq by Expectation Maximization normalized expression data for the TCGA HNSC cohort was extracted and analyzed as described previously [23]. Briefly, primary patient samples with known HPV status were manually grouped as HPV+, HPV-, or normal-adjacent control tissue based on other previously published datasets [21,35]. In these datasets, HPV status was assigned by aligning RNA-seq reads for the HPV oncogenes expressed in the tumours to the different high risk HPV types. This resulted in 73 HPV+, 442 HPV-, and 43 normal-adjacent control samples with data available for gene expression comparisons. Note that all HPV+ samples had high risk HPV as follows: HPV16 (61 samples), HPV33 (8 samples), HPV35 (3 samples), and HPV56 (1 sample). The five-number summary, mean, and pairwise statistical tests were calculated using R (version 3.4.0) for all 229 metabolic genes analyzed (see Supplementary Table S1). These 229 genes were manually selected (Supplementary Table S1) as they are involved in eight central cellular metabolic pathways or processes, of which cellular respiration was then separated into its respective complexes. The number of genes examined from each pathway was as follows: glycolysis, 36 genes (adapted from GO:0061621 and [55]); TCA cycle, 20 genes (adapted from GO:0006099 and [56]); mitochondrial respiratory complex I, 43 genes (adapted from GO:0045333, GO:0046043 and [57]); mitochondrial respiratory complex II, 4 genes (adapted from GO:0045333, GO:0046043 and [58]); mitochondrial respiratory complex III, 9 genes (adapted from GO:0045333, GO:0046043 and [59,60]); mitochondrial respiratory complex IV, 31 genes (adapted from GO:0045333, GO:0046043 and [61]); mitochondrial ATPase, 16 genes (adapted from GO:0046043 and [62]); fatty acid synthesis, 21 genes (adapted from GO:0019368, GO:0046949, GO: 0006629 and [63,64,65,66]); β-oxidation, 18 genes (adapted from GO:0003995, GO:0003985, GO: 0004300 and [67]); glutaminolysis, 7 genes (adapted from GO:0004069, GO:0004352, GO: 0004359, GO: 0004021 and [68]); pentose phosphate pathway, 11 genes (GO:0006098 and [69]); monocarboxylic acid transport (MCT) family, 13 genes (adapted from GO:0008028 and [15]). Boxplot comparisons of gene expression were made with GraphPad Prism v7.0 (Graphpad Software, Inc., San Diego, California, USA). For the boxplots, center lines show the medians, box limits indicate the 25th and 75th percentiles, and whiskers extend 1.5 times the interquartile range from the 25th and 75th percentiles. P-values were assigned using a two-tailed non-parametric Mann-Whitney U test using Graphpad Prism. Bivariate analysis for selected genes was performed through R (version 3.4.0) using the Spearman rank correlation coefficient. The proportion of genes in each pathway that were up or downregulated among each comparison was represented in a bar graph and was calculated as follows: number of genes upregulated (or downregulated) in a comparison (e.g., HPV+ HNSCC vs HPV- HNSCC) divided by the total number of genes in that pathway. The proportion of downregulated genes were represented as a negative value.

### 4.3. Survival Analysis

Five-year overall survival outcomes were compared in both HPV+ and HPV- subsets of HNSCC patients dichotomized by median expression for all metabolic genes listed in Supplementary Table S1. Log-rank statistical p-values were calculated for each Cox survival model. The derived log-rank p-values for all tested genes (listed in Supplementary Table S2) were assessed for significance after correcting for false discovery rate (FDR) using the Benjamini–Hochberg method, and an FDR threshold of 0.1 was set for significance. Univariate analysis was performed through R (version 3.4.0) based on a Cox Proportional Hazard Model using the survival package (version 2.41-3). Stepwise bidirectional multivariate analysis was then carried out with clinical variables (sex, age, subsite, T stage, N stage, Overall stage, and HPV type), and *SDHC*, *COX7A1*, *COX16*, *COX17*, *ELOVL6*, *GOT2*, and *SLC16A2* expression—low expression of these 7 genes were found to be statistically correlated with improved survival after univariate analysis. The p-values derived from the Wald test on survival coefficients were reported for investigated variables. Furthermore, a second set of survival outcomes were determined to compare HPV+ tumours expressing low levels of each combination of genes that were significantly correlated with improved survival after multivariate analysis—*COX16*, *COX17*, and *SLC16A2*.

### 4.4. Gene Enrichment Analysis

We performed a gene enrichment analysis on our seven survival-associated genes using the Go Enrichment Analysis feature on http://geneontology.org [70,71]. This analysis is powered by PANTHER14.1 (PANTHER Overrepresentation Test) using the “GO cellular component complete” annotation data set with a Fisher’s exact test followed by a calculation of false discovery rate (cutoff = FDR P < 0.05) to determine statistical significance [72].

### 4.5. Analysis of Differential Cell Line Sensitivity to Tyrosine Kinase Inhibitors Based on HPV Status

B-scores (mean ± SEM) reflecting drug activity were extracted from a previously conducted high throughput drug screen using 27 HNSCC cell lines [44]. The average B-scores for the indicated tyrosine kinase inhibitors (TKIs) was calculated for the 6 HPV+ and 21 HPV- HNSCC lines and plotted (Supplementary Figure S2).

## 5. Conclusions

In summary, our analysis of HNSCC TCGA data stratified by HPV status indicated that the metabolic profile of HPV+ and HPV- HNSCC are strikingly different. HPV- HNSCCs may utilize glycolysis to a greater extent than HPV+ HNSCCs, while HPV+ HNSCCs may be more reliant on the TCA cycle, cellular respiration, and β-oxidation than HPV- HNSCCs. Despite this difference, both types of HNSCCs likely exhibit far less cellular respiration than normal head and neck tissues, consistent with a cancer-associated Warburg phenotype [73]. Importantly, expression of genes involved in mitochondrial complex II and mitochondrial complex IV were associated with survival for HPV+ HNSCC patients. Namely, low expression of *SDHC*, *COX7A1*, *COX16,* or *COX17* was associated with better survival outcomes. Low expression of *ELOVL6*, involved in fatty acid elongation; *GOT2*, involved in amino acid metabolism; and *SLC16A2*, involved in thyroid hormone transport, were also all associated with better survival outcomes in HPV+ HNSCC patients. However, of these genes, only *COX16*, *COX17* and *SLC16A2* were independently correlated with survival outcomes according to our multivariate analysis. Importantly, *COX16*, *COX17*, and *SLC16A2* were associated with near 100% survival in all patients with low expression of any two of these genes. The products of these genes may represent useful new therapeutic targets for HPV+ HNSCC, as inhibition of their functions could phenocopy the metabolism of those tumours with low levels of metabolic gene expression, leading to improved survival in HPV+ HNSCC patients.

## Figures and Tables

**Table 1:** Univariate and multivariate analysis of the association of clinical variables and expression of metabolic genes with overall survival in HPV+ HNSCC.

Variables		Univariate		Multivariate	
		HR (95% CI)	P value	HR (95% CI)	P value
Sex	Male vs Female	0.81 (0.18–3.62)	0.78		
Age	per every additional year	0.99 (0.94–1.04)	0.74		
	Oral cavity vs Oropharynx	2.82 (1.02–7.80)	**0.045**	13.59 (2.67–69.11)	**0.002**
Subsite	Larynx vs Oropharynx	1.52 x 10^−8^ (0–Inf)	1.00	3.67 x 10^−10^ (0–Inf)	1.00
	Hypopharynx vs Oropharynx	1.57 x 10^−8^ (0–Inf)	1.00	1.10 x 10^−8^ (0–Inf)	1.00
T Stage	T3–T4 vs T1–T2	1.03 (0.36–2.91)	0.96		
N Stage	N2b–N3 vs N0–N2a	0.41 (0.14–1.19)	0.10		
Overall Stage	IV vs I–III	0.76 (0.26–2.24)	0.62		
HPV Type	33, 35, 56 vs 16	3.33 (1.14–9.78)	**0.028**	16.88 (3.24–87.88)	**0.0008**
*COX16*	Low vs High Expression	0.19 (0.05–0.72)	**0.015**	0.059 (0.009–0.39)	**0.003**
*COX17*	Low vs High Expression	0.13 (0.03–0.47)	**0.002**	0.03 (0.003–0.35)	**0.005**
*COX7A1*	Low vs High Expression	0.22 (0.06–0.77)	**0.018**	0.25 (0.04–1.56)	0.14
*ELOVL6*	Low vs High Expression	0.17 (0.04–0.65)	**0.009**		
*GOT2*	Low vs High Expression	0.24 (0.07–0.79)	**0.019**		
*SDHC*	Low vs High Expression	0.23 (0.07–0.77)	**0.018**		
*SLC16A2*	Low vs High Expression	0.17 (0.04–0.63)	**0.008**	0.07 (0.01–0.37)	**0.002**

## Supplementary Materials

The following are available online at https://www.mdpi.com/2072-6694/12/1/253/s1, Figure S1: Correlation plots of independently significant genes identified by multivariate analysis, Figure S2: Tyrosine kinase inhibitor activity in 27 HNSCC cell lines based on HPV status, Table S1: Metabolic pathway gene expression, Table S2: Survival curve statistics.
