# Supplementary material for: Survival-Associated Metabolic Genes in Human Papillomavirus-Positive Head and Neck Cancers
Source: Cancers (Basel). 2020 Jan 20;12(1):253. doi: 10.3390/cancers12010253 (PMC7017314; doi:10.3390/cancers12010253)
Supplement: Supplementary file 1 [file cancers-12-00253-s001.zip › cancers-668063-supplementary material/Supplementary Figure S2 - Tyrosine kinase inhibitor activity in 27 HNSCC cell lines based on HPV status.pdf]

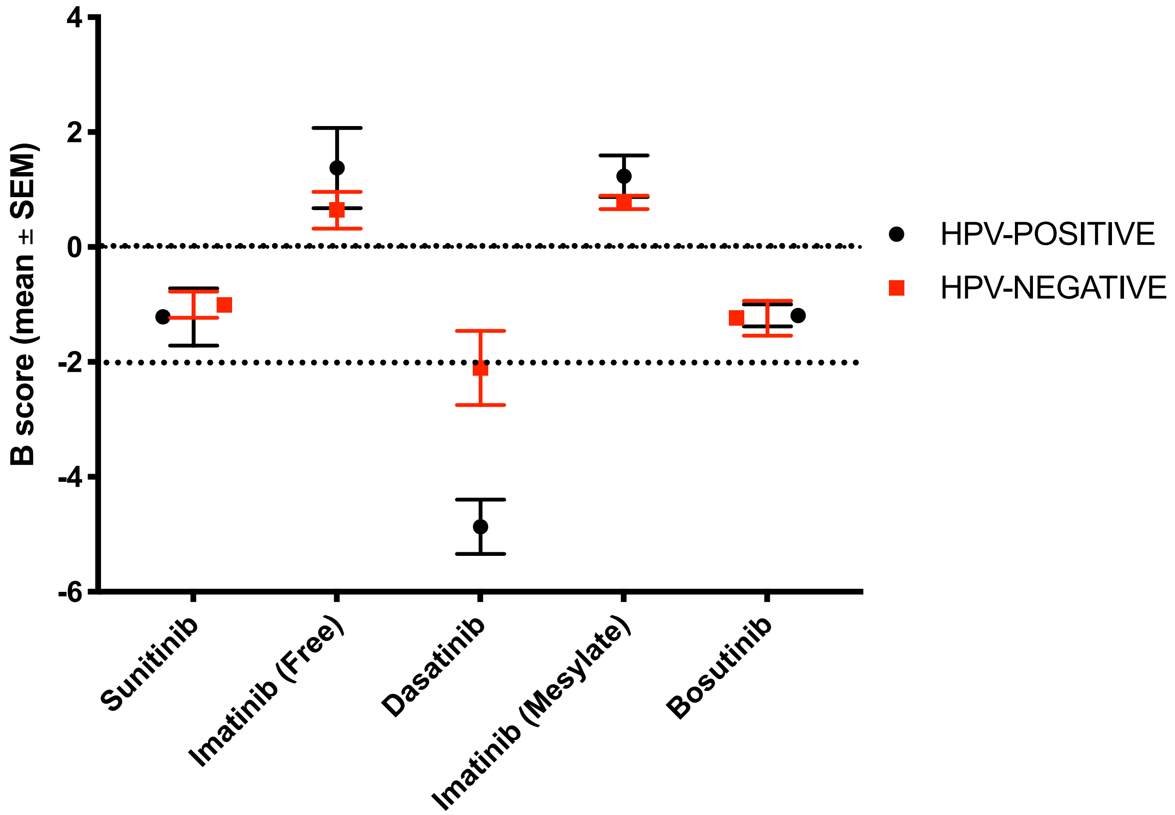

Supplementary Figure 1. Tyrosine kinase inhibitor activity in 27 HNSCC cell lines based on HPV status. B-scores +/- SEM were calculated for HPV+ and HPV- HNSCC cell lines from a high throughput drug screen. Active drugs exhibit a B-score below -2. Red line = HPV-, Black line = HPV+.
