# Supplementary material for: Survival-Associated Metabolic Genes in Human Papillomavirus-Positive Head and Neck Cancers
Source: Cancers (Basel). 2020 Jan 20;12(1):253. doi: 10.3390/cancers12010253 (PMC7017314; doi:10.3390/cancers12010253)
Supplement: Supplementary file 1 [file cancers-12-00253-s001.zip › cancers-668063-supplementary material/Supplementary FigureS1 - Correlation plots of independently significant genes identified by multivariate analysis.pdf]

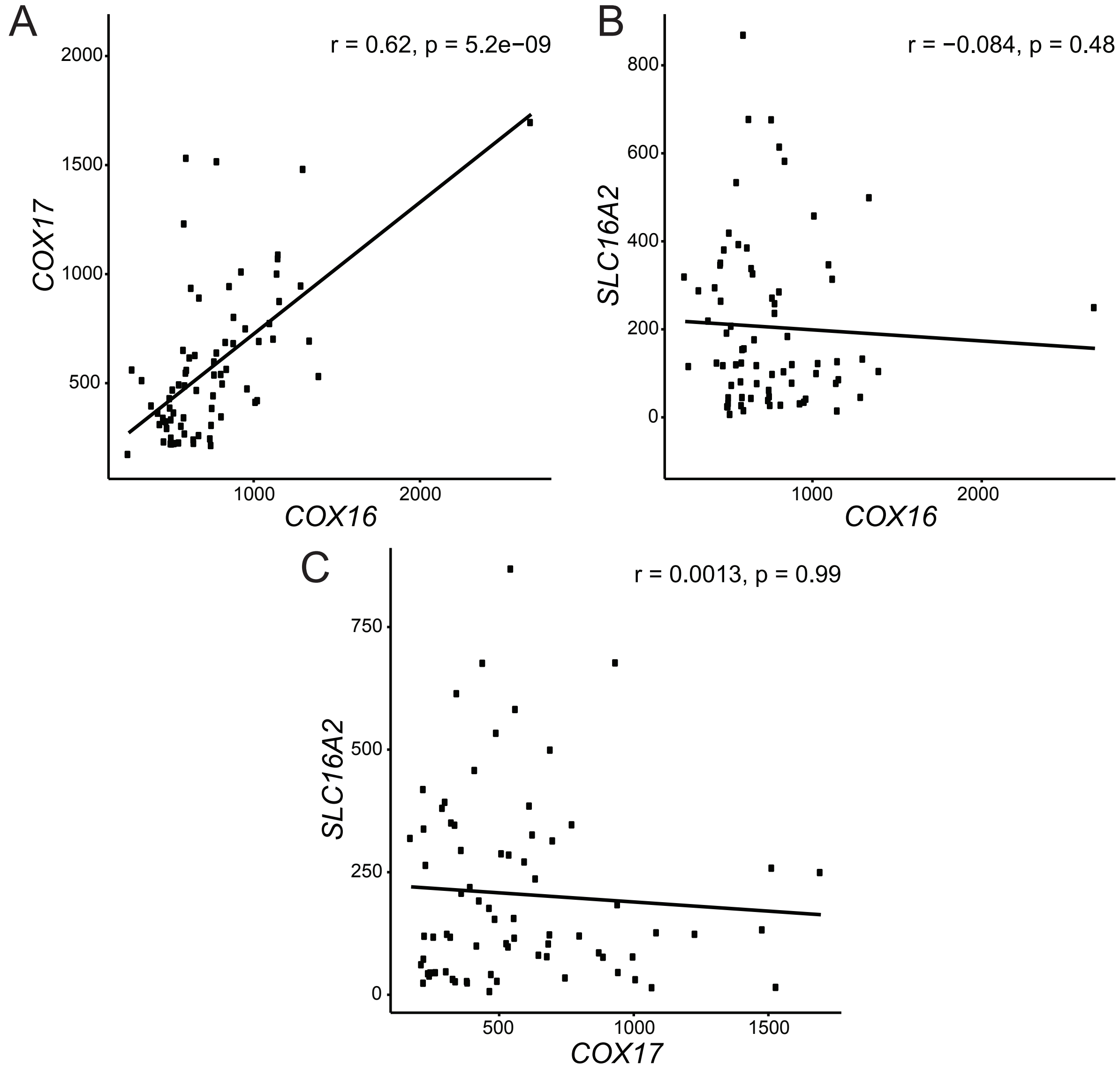

Supplementary Figure 2. Correlation plots of independently significant genes identified by multivariate analysis. (**A**) Expression *COX16* and *COX17* were significantly correlated (r=0.62, p=5.2 x 10^{-9}) in HPV+ HNSCCs. (**B**) Expression *SLC16A2* and *COX16* were not correlated (r =-0.084, p=0.48). (**C**) Expression of *SLC16A2* and *COX17* were not correlated (r=0.0013, p=0.99).
